# Comparison of the Bond Strength of Primary Molar Bands to Zirconia and Stainless‐Steel Crowns Following Sandblasting and Application of Different Cements

**DOI:** 10.1155/ijod/6418886

**Published:** 2026-08-19

**Authors:** Ladan Pakzad, Ezatollah Jalalian, Ali Rashidian

**Affiliations:** ^1^ Department of Pediatric Dentistry, Tehran Dental Branch, Tehran Medical Science, Islamic Azad University, Tehran, Iran, ctb.iau.ir; ^2^ Department of Prosthodontic, Tehran Dental Branch, Tehran Medical Science, Islamic Azad University, Tehran, Iran, ctb.iau.ir

**Keywords:** air abrasion, crowns, dental, orthodontic, space maintenance, stainless steel, zirconium oxide

## Abstract

**Objectives:**

This study compared the bond strength of primary molar bands to zirconia crowns (ZCs) and stainless‐steel crowns (SSCs) following sandblasting and application of glass ionomer (GI) and self‐adhesive resin cements.

**Materials and Methods:**

This in vitro study was conducted on 48 monolithic ZCs and 48 primary mandibular second molar SSCs mounted in auto‐polymerizing acrylic resin in cylindrical molds. Orthodontic wire was used to create a 70‐mm U‐shaped loop soldered to the mid‐buccal and mid‐lingual surfaces perpendicular to the occlusal surface. The specimens were assigned to 8 groups for the bonding of sandblasted and non‐sandblasted bands to SSCs and ZCs with GI and self‐adhesive resin cement. After the interventions and incubation in artificial saliva at 37°C for 24 h, the specimens were thermo‐cycled (5000 cycles), and their bond strength was measured in a universal testing machine (1 mm/minute). Data were analyzed by three‐way ANOVA (alpha = 0.05).

**Results:**

The bond strength was the highest in the sandblasted band bonded to SSC with resin cement and the lowest in the non‐sandblasted band bonded to ZC with GI. Irrespective of crown type, resin cement yielded a significantly higher bond strength than GI (*p*  < 0.001). Also, irrespective of crown and cement type, sandblasting significantly increased the bond strength (*p*  = 0.004).

**Conclusion:**

The bond strength provided by resin cement was significantly higher than GI cement. Sandblasting of the band significantly increased the bond strength, but the effect of crown type on the bond strength was not significant.

## 1. Introduction

The bond strength provided by cementation of orthodontic bands to primary molars should be sufficiently high to withstand forces in the oral environment [1]. Evidence shows that failure of the band often occurs at the cement‐band interface, compromising the treatment success [2]. Also, bonding of the band to stainless steel crowns (SSCs), zirconia crowns (ZCs), extensively carious abutment teeth, and those with a history of pulp therapy is complicated and challenging [3]. Orthodontic bands are part of fixed space maintainers to preserve the integrity of dental arch following early loss of primary molars. Thus, in case of failure and debonding of the band, teeth adjacent to the edentulous space may undergo mesial and distal tipping and lead to space loss. Subsequently, crowding, ectopic eruption of permanent successors, or even their impaction may occur [4, 5].

Several cements may be used for the bonding of orthodontic bands. Previously, zinc phosphate cement was extensively used for this purpose. However, its application has been limited due to its high solubility and complete reliance on mechanical adhesion [6]. Polycarboxylate cement chemically reacts with enamel and steel; however, it also has limited application due to shortcomings such as high viscosity, short setting time, and high solubility [7]. Glass ionomer (GI) cement has less solubility and higher tensile and compressive strength than zinc phosphate cement [8]. However, high moisture sensitivity during the setting reaction is a drawback of GI cement [9], which led to the introduction of resin‐modified GI with higher physical properties [9].

Self‐adhesive resin cements are the newest generation of cements with the optimal properties of previous generations. They provide micromechanical interlocking and chemical retention that create a sufficiently high bond strength to enamel [10]. Kalaskar et al. [11] compared the bond strength of orthodontic bands to SSCs and ZCs using GI and self‐adhesive resin cement and showed that self‐adhesive resin cement yielded a higher bond strength to both SSCs and ZCs. Sandblasting of the internal surface of the band has also been recommended to decrease the oxide layer and increase the bonding surface area and enhance the bond strength as such [12]. Bawazir et al. [7] reported that sandblasting of the bands increased their bond strength irrespective of cement type.

Considering the scarcity of studies on the bond strength of primary molar bands to SSCs and particularly ZCs and lack of studies on the effects of crown type, cement type, and sandblasting of the band altogether in this regard, this study aimed to compare the bond strength of primary molar bands to ZCs and SSCs following sandblasting and application of GI and self‐adhesive resin cement.

## 2. Materials and Methods

This in vitro experimental study was conducted on #33 and #34 SS bands (Shinhung, South Korea), #4 primary mandibular right second molar SSCs (Shinhung, South Korea), and #4 primary mandibular right second molar ZCs (NU Smile, USA). The study protocol was approved by the ethics committee of the university (R.IAU.DENTAL.REC.1402.113).

### 2.1. Sample Size

The sample size was calculated to be 12 in each group according to a study by Kalaskar et al. [11], using fixed effect ANOVA power analysis of PASS 11 assuming *α* = 0.05, and study power of 0.99, 0.97, and 0.95 for independent variables each with 2 surfaces and effect sizes of 0.474, 0.395, and 0.368, respectively.

### 2.2. Specimen Preparation

**Figure 1:**
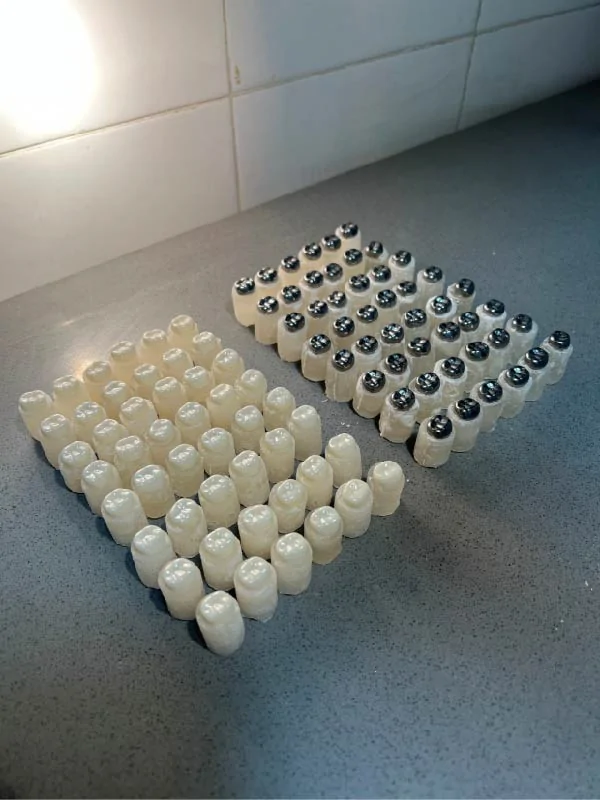
Mounted samples.

**Figure 2:**
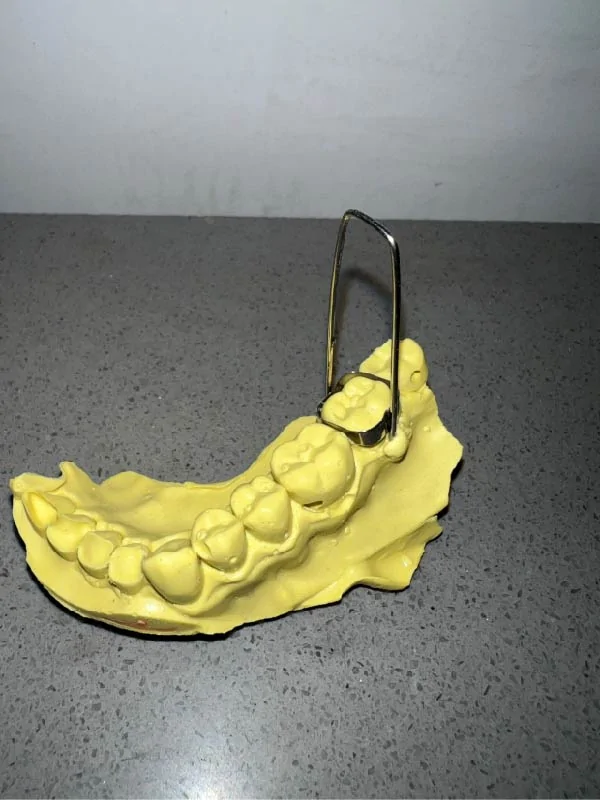
Preparing orthodontic U‐shaped wire to solder to the occlusal surface of the band.

**Figure 3:**
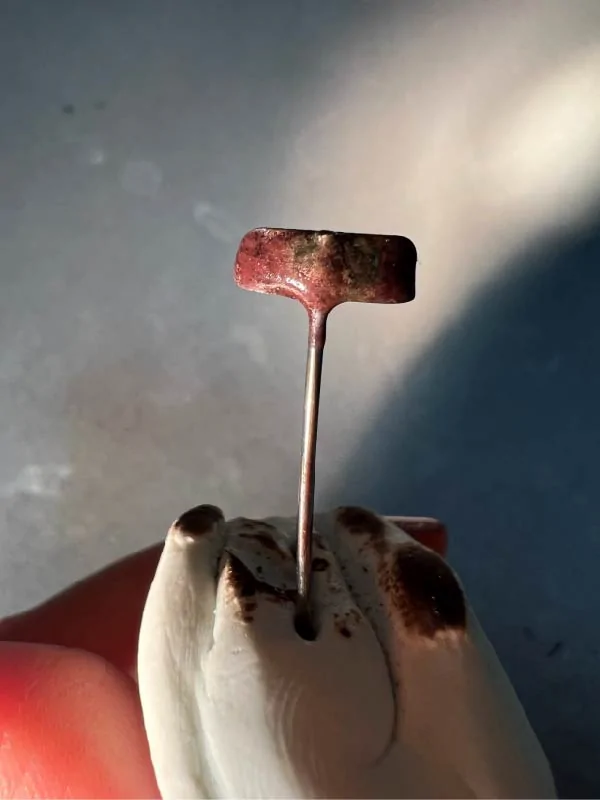
Appearance of soldered band before electro‐polishing.

**Figure 4:**
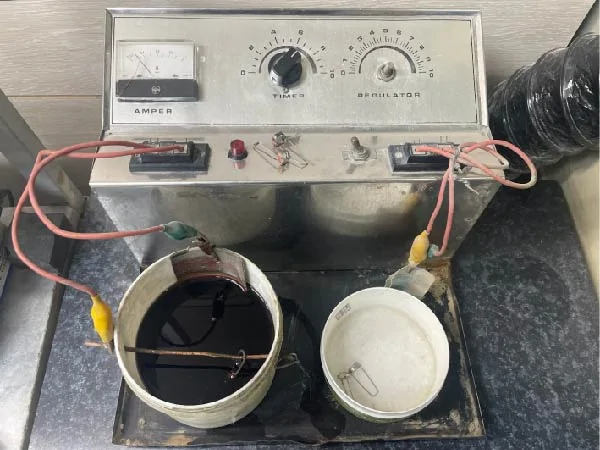
Placing soldered bands in acidic bath of electro‐polisher.

**Figure 5:**
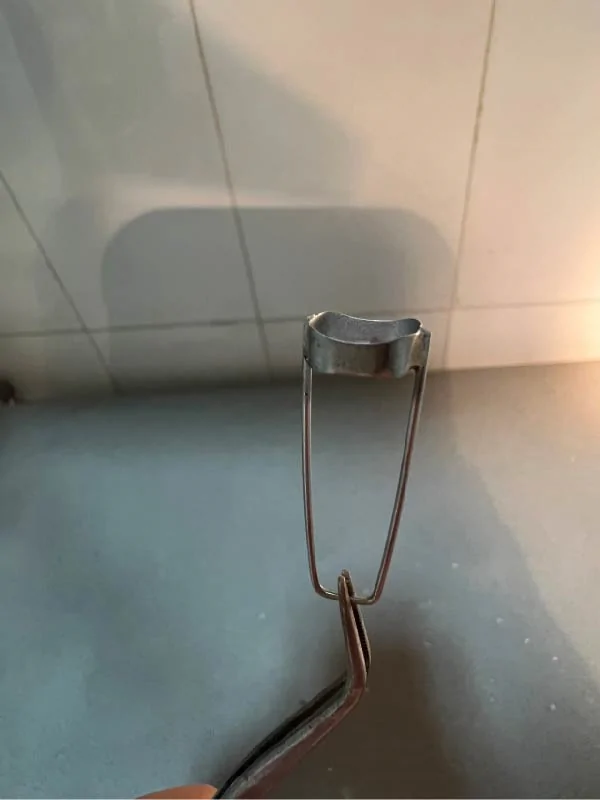
Appearance of soldered band after electro‐polishing.

**Figure 6:**
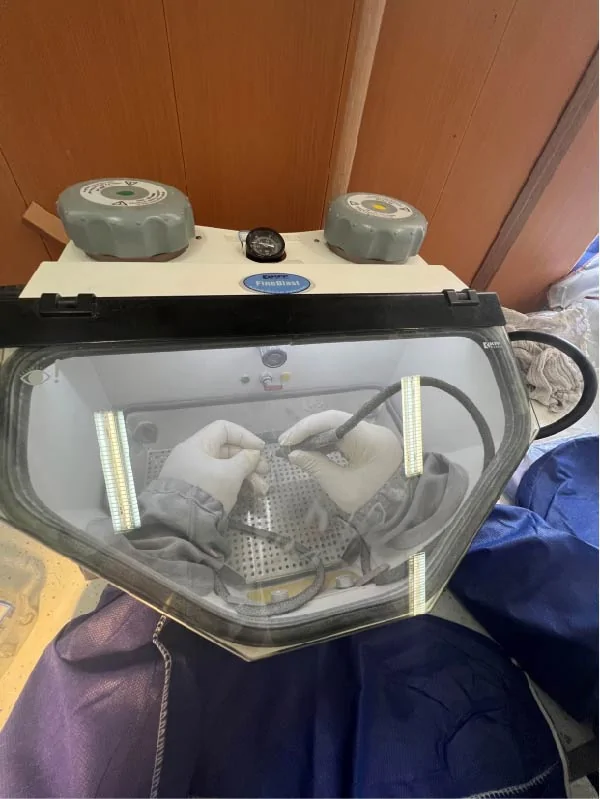
Sandblasting the internal surface of bands.

**Figure 7:**
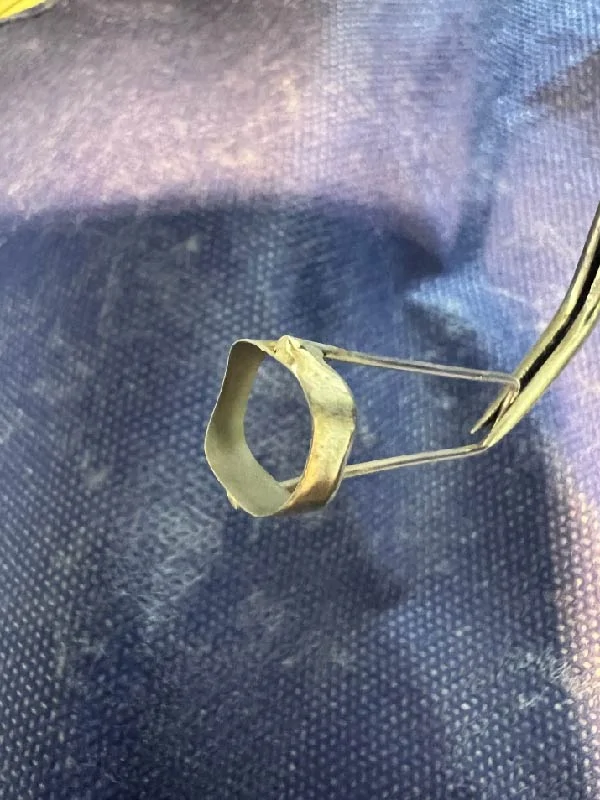
Appearance of the internal surface of the band after sandblasting.

A total of 48 monolithic ZCs and 48 SSCs of primary mandibular right second molars were used for this study. They were filled with auto‐polymerizing acrylic resin (Acrosun, Iran) to 0.5 mm to their cervical margin and were then mounted in cylindrical molds measuring 20 × 40 mm filled with auto‐polymerizing acrylic resin along their longitudinal axis. Next, a 19‐gauge (0.9 mm) orthodontic wire (American Orthodontics, USA) was used to create a 70‐mm U‐shaped loop, which was soldered to the mid‐buccal and mid‐lingual surfaces perpendicular to the occlusal surface (Figures 1–3); #34 bands were used for ZCs, and #33 bands were used for SSCs. All soldered bands were placed in the acidic bath of an electro‐polisher (MSPCO, Iran) to eliminate flux and oxide layers. Half of the bands were sandblasted (Fine Blast, KFP Co., Iran) with 50 µm aluminum oxide particles under 2.8 bar pressure at 10 mm distance perpendicular to each of the buccal, lingual, and mesial surfaces for 10 s (Figures 4–7) [13].

In this study, random number tables were utilized for the allocation of samples to their respective subgroups. The universal testing machine operator and outcome assessor were blind to this allocation. Ultimately, the specimens were randomly assigned to 8 groups (*n* = 12), according to the type of crown and cement and conduction of sandblasting, as follows:

Group 1. Sandblasted primary molar bands were bonded to SSCs by using GI cement (GC Fuji I, Japan).

Group 2. Non‐sandblasted primary molar bands were bonded to SSCs by using GI cement.

Group 3. Sandblasted primary molar bands were bonded to SSCs by using self‐adhesive resin cement (Duo Link, Bisco Co., USA).

Group 4. Non‐sandblasted primary molar bands were bonded to SSCs by using a self‐adhesive resin cement.

Group 5. Sandblasted primary molar bands were bonded to ZCs using GI cement.

Group 6. Non‐sandblasted primary molar bands were bonded to ZCs by using GI cement.

Group 7. Sandblasted primary molar bands were bonded to ZCs by using a self‐adhesive resin cement.

Group 8: Non‐sandblasted primary molar bands were bonded to ZCs by using a self‐adhesive resin cement.

### 2.3. Cementation

Cement was applied on the internal surface of the band, and the band was placed on the respective specimen with hand pressure and using a band pusher. For this purpose, cement was prepared according to the manufacturer’s instructions. As instructed by Bisco for cementation of ZCs with Duo Link self‐adhesive resin cement, zirconia cleanser (Zr clean, Bisco, USA) was used prior to cementation in order to decrease surface contaminations and increase the surface energy of the substrate. Next, a zirconia primer (Z‐Prime, Bisco, USA) was applied on the internal surface to create a chemical bond between the zirconia and cement [14, 15]. As instructed by the manufacturer, Zr Clean was applied on all ZC surfaces for 20 s, and was then washed with air/water spray. Subsequently, one layer of Z‐Prime was applied on the specimen by a micro‐brush, and after 30 s, the band with cement was placed over the specimens. Finally, excess cement was removed from the occlusal and cervical surfaces by a dry cotton roll.

**Figure 8:**
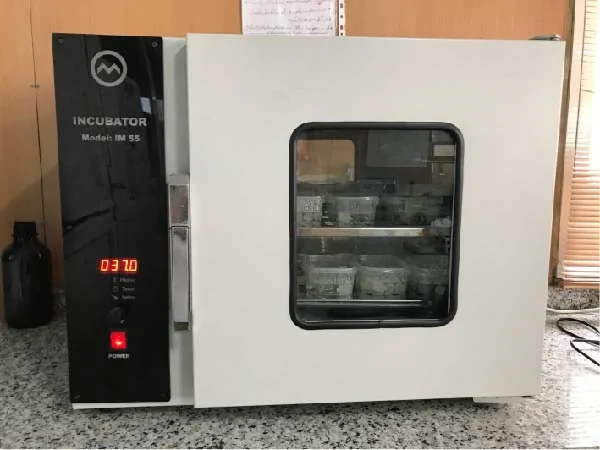
Incubating immersed specimens in artificial saliva at 37°C for 24 h.

In self‐adhesive resin cement groups, the crowns were cemented and cured for 20 s using a LED curing unit (LED‐D, Woodpecker) with a light intensity of 1200 mW/cm^2^ [11]. After the complete setting of the cement, the specimens were immersed in artificial saliva and incubated at 37°C for 24 h (Figure 8).

### 2.4. Thermo‐Cycling

**Figure 9:**
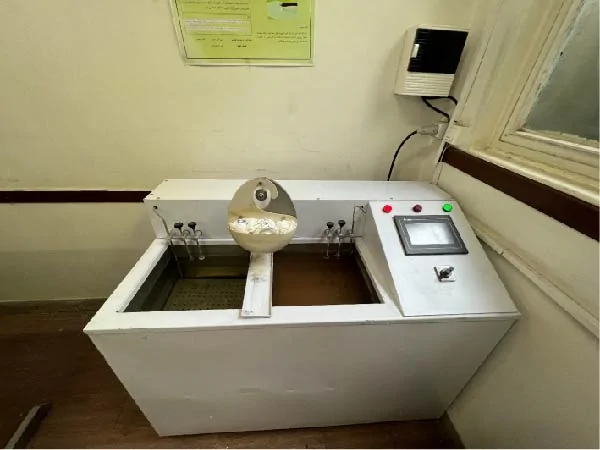
Thermo‐cycling specimens under 5000 thermal cycles between 5 and 55°C.

The specimens underwent 5000 thermal cycles between 5 and 55°C with a dwell time of 20 s and a transfer time of 10 s (Figure 9) [16].

### 2.5. Bond Strength Measurement

**Figure 10:**
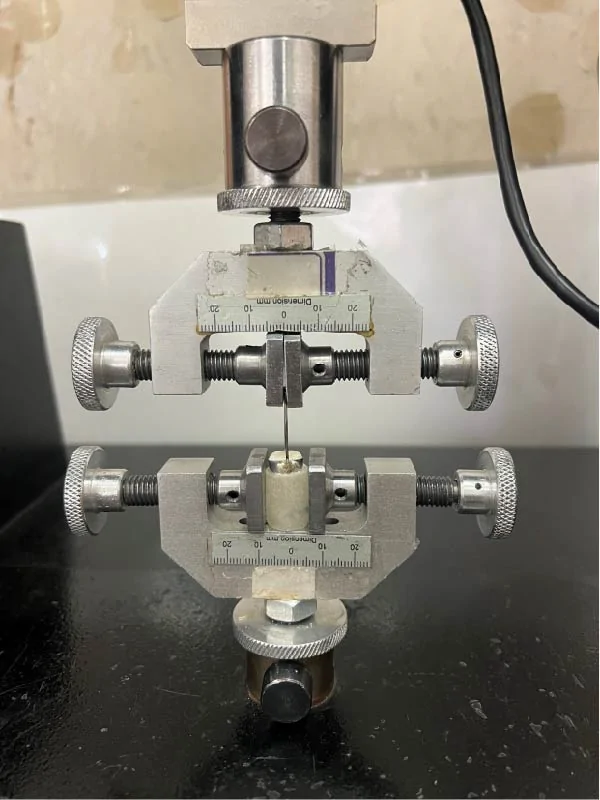
Placement of the sample in the UTM for measuring bond strength.

The bond strength was measured by a blind operator toward the study groups in a universal testing machine (Santam Co., Iran) at a crosshead speed of 1 mm/minute. The vertical distance between the upper and lower compartments was the same and equal to 4 cm for all specimens. The mounted specimens were connected to the lower compartment from the acrylic part and to the upper compartment through the U‐shaped wire loop such that the loop was in line with the longitudinal axis of the crown and band and perpendicular to the occlusal surface. Accordingly, the load was applied perpendicular to the occlusal surface in the pull‐out mode, and the magnitude of load required for detachment of the band from the crown surface was recorded in Newton (N) (Figure 10).

To measure the bond strength in megapascal (MPa), the load was divided by the surface area of the band, which was calculated by multiplying the length (occlusogingival height) of the band in millimeters by the band periphery (pi number multiplied by the band diameter) in square millimeters [8].

### 2.6. Statistical Analysis

Data were analyzed by three‐way ANOVA using SPSS version 25 (SPSS Inc., IL, USA) at the 0.05 level of significance.

## 3. Results

Table 1 presents the measures of central dispersion for the bond strength in the 8 groups. As shown, the highest bond strength belonged to the sandblasted band bonded to SSC with self‐adhesive resin cement, and the lowest was recorded in the bonding of non‐sandblasted band to ZC using GI cement. Three‐way ANOVA showed no significant difference in bond strength among the interaction effects between tested variables (restoration type  ^∗^ cement type, restoration type ^∗^sandblasting, and cement type ^∗^sandblasting) (*p*  = 0.859). The two‐way interaction effects of restoration type and cement type (*p*  = 0.927), restoration type and sandblasting (*p*  = 0.492), and cement type and sandblasting (*p*  = 0.952) on bond strength were not significant.

**Table 1:** Measures of central dispersion for the bond strength (MPa) in the eight groups.

Group	Crown type	Cement type	Sandblasting	Mean	SD	95% CI	
						Lower bound	Upper bound
1	SSC	GI	Yes	1.4693	0.07837	1.4195	1.5191
2	SSC	GI	No	1.4289	0.09246	1.3702	1.4877
3	SSC	Resin cement	Yes	1.7831	0.10524	1.7162	1.8500
4	SSC	Resin cement	No	1.7466	0.09610	1.6856	1.8077
5	ZC	GI	Yes	1.4553	0.03341	1.4341	1.4765
6	ZC	GI	No	1.3979	0.07186	1.3522	1.4435
7	ZC	Resin cement	Yes	1.7720	0.08399	1.7186	1.8253
8	ZC	Resin cement	No	1.7066	0.07084	1.6616	1.7516
			Total	1.5950	0.17870	1.5587	1.6312

Comparison of the effect of each parameter (restoration type, cement type, and sandblasting) alone on bond strength (Table 2) revealed that the bond strength in resin cement groups was significantly higher than that in GI groups (*p*  < 0.001). Also, the bond strength in sandblasted groups was significantly higher than that in non‐sandblasted groups (*p*  = 0.004). However, restoration type had no significant effect on the bond strength (*p*  = 0.152). Also, the cement type had a greater effect on bond strength than sandblasting of the band.

**Table 2:** Comparison of the effect of restoration type, cement type, and sandblasting on bond strength.

Parameter	Category	Mean	SD	*p*‐Value ^∗^
Restoration type	SSC	1.6069	0.092	0.152
	ZC	1.5829	0.064	
Cement type	GI	1.4378	0.068	≤0.001
	Resin cement	1.7520	0.088	
Sandblasting	Yes	1.6199	0.074	0.004
	No	1.5700	0.082	

^∗^Three‐way ANOVA.

## 4. Discussion

This study compared the bond strength of primary molar bands to ZCs and SSCs following sandblasting and application of GI and self‐adhesive resin cements. The results showed that irrespective of the crown type, self‐adhesive resin cement yielded a significantly higher bond strength than GI cement. Also, irrespective of crown type and cement type, sandblasting significantly increased the bond strength. Thus, a higher bond strength may be achieved between the space maintainer band and SSCs and ZCs by sandblasting of the band and using a self‐adhesive resin cement.

Srivastava et al. [3] compared the efficacy of resin cement and GI for the cementation of SS bands to molar teeth. They showed that resin cement yielded the lowest micro‐leakage and the highest bond strength, which was in agreement with the present results. Also, Elsoud et al. [17] reported that resin cement in the presence/absence of saliva yielded a higher bond strength than GI cement.

Self‐adhesive resin cements can bond to tooth structure without requiring separate application of adhesive or etchant. The main components of self‐adhesive resin cements include acidic functional monomers such as 10‐MDP, conventional dimethyl methacrylate monomers (such as bis‐GMA), fillers, and activator‐initiator systems [18]. Similar to self‐etch adhesives, functional acidic monomers create a low‐pH environment with high hydrophilicity during the initial setting reaction, which enables the material to wet and etch the tooth surface and enhances homogeneous penetration of adhesive resin [19]. As the reaction progresses, acidic functional groups react with the calcium in tooth structure and metal oxides of inorganic fillers with ionizing ability, leading to a pH rise and hydrophobicity [20].

In the current study, restoration type (SSC/ZC) had no significant effect on the bond strength. Kalaskar et al. [11] compared the bond strength of SS bands to SSCs and ZCs using resin cement and GI. They reported that irrespective of restoration type, resin cement yielded the highest bond strength in all groups. Alrashdi et al [21], in their review study, reported the success rate of bonding of GI cement to ZC of primary teeth to be 93%. Also, Bawazir et al. [7] assessed the bond strength of SS bands to SSCs using different cements. They demonstrated that polycarboxylate and GI cements yielded a significantly higher bond strength than GI. GI cement is prepared by mixing its powder containing calcium fluoroaluminosilicate with its liquid composed of polycarboxylic acid in water [22]. After mixing of the powder and liquid, GI setting reactions are initiated through acid–base reactions by the formation of ionic bonds between the carboxyl groups of polycarboxylic acid and calcium and aluminum ions of the powder [23]. GI can chelate calcium and phosphate ions and form molecular adhesion to enamel and dentin as such [18]. Fluoride release, biocompatibility with dental pulp, and a coefficient of thermal expansion close to that of tooth structure are among the advantages of GI cement. However, it has several drawbacks such as a high modulus of elasticity, susceptibility to bulk fracture, high solubility in the oral environment, and subsequent micro‐leakage [24]. GI can also form ionic bonds to metal restorations, bands, and SSCs [2]. An in vitro study reported that the bond strength of GI cement to enamel was higher than its bond strength to SS orthodontic bands because the band‐cement interface is more susceptible to fracture, and residual cement remains attached to enamel. Thus, the bond strength of SS orthodontic bands to SSCs is not expected to be as high as the bond strength of the band to enamel [7].

Özcan and Bernasconi [25] in their meta‐analysis assessed the adhesion potential of resin cements and GI to zirconia and the influential factors in this respect. They concluded that physicochemical preparation of the zirconia surface and application of resin cements with an MDP base may result in higher bond strength in macro‐ and micro‐tensile tests. It has been emphasized that MDP‐based resin cements can form a strong bond to zirconia [26], and such a high bond strength has been attributed to the chemical reaction of MDP with zirconium oxide [26]. This chemical reaction has been confirmed by secondary ion mass spectrometry and Fourier‐transform infrared spectroscopy [27, 28].

Prefabricated ZCs for primary teeth are glazed by the manufacturer. Thus, the chemical reaction of cement with the glazed zirconia surface is not feasible to benefit from the maximum potential of resin cements in the provision of a high bond strength. The glaze layer may be eliminated by a bur to expose the zirconia surface. However, this technique has some limitations. Contact of bur with the zirconia surface can lead to the formation of microcracks and may compromise the final strength of restoration. Also, the created rough zirconia surface can cause wear of the opposing teeth, and enhance plaque accumulation, leading to gingival inflammation and periodontal disease [29]. Kheur et al. [30], in their in vitro study, indicated that bur preparation and polishing of monolithic zirconia surface could not return the initial smoothness, and not only the surface roughness but also the hardness and tensile strength of restoration are affected by bur preparation and polishing. Sandblasting can also be performed for elimination of the glaze layer. Although this technique can increase the surface area and micromechanical retention, it adversely affects the physical properties and can lead to crack formation [31, 32]. Elimination of the glaze layer was not performed in the present study due to maximum simulation of the clinical setting. By retaining the glaze layer on Zr crowns, there is a possibility that the adhesive potential of zirconia may have been underestimated, which can affect generalizability.

The present results showed the superiority of self‐adhesive resin cement to GI in terms of bond strength. The main advantage of the GI cement is its fluoride release, which can prevent caries when the band is directly placed on molar teeth [18]. However, this property does not appear to be important when the band is placed on a SSC or ZC. Also, Duo‐Link is a translucent cement with low film thickness, which enhances placement of the band on the crowns for cementation and improves esthetics. Furthermore, its tube has an auto‐mix tip, which saves time and prevents manual mixing errors and their adverse effects on the bond strength and physical properties of the cement [33].

Sandblasting of the internal surface of the band decreases the oxide layer and increases the bonding surface area, resulting in improved micromechanical interlocking and higher bond strength [12]. Sandblasting also increases the surface energy and surface wetting [34, 35]. Bawazir et al. [7] reported that sandblasting of the bands increased their bond strength irrespective of the cement type. Nalawade et al. [36] indicated that sandblasting of the bands significantly increased the bond strength of GI and zinc phosphate cements by 2‐folds and zinc polycarboxylate cement by more than 2‐folds. Kalim and Mitali [37] demonstrated that sandblasting significantly increased the bond strength of orthodontic bands. The present results also showed that sandblasting increased the bond strength in use of both GI and resin cement. Veerabadhran et al. [38] showed no significant effect of sandblasting on bond strength of primary molar SSCs, which was in contrast to the present results and the available literature in this respect.

This study had several strengths. The specimens were stored in artificial saliva at the oral environment temperature (37°C) and underwent thermo‐cycling to better simulate the oral clinical environment. Also, the effect of a number of parameters with possible influential effects on bond strength, including the crown type, cement type, and sandblasting was evaluated.

Furthermore, the results of the study provide practical guidance for clinicians working with prefabricated ZCs by utilizing self‐adhesive cement and sandblasting to enhance bond strength.

This study had limitations as well. Due to the recent introduction of ZCs to pediatric dentistry, not many studies are available regarding the bond strength of SS bands to ZCs [15] to compare our results with. Also, this study had an in vitro design, which limits the generalizability of the findings to the clinical setting. Future clinical trials are required to obtain more reliable results. As well, the findings are most directly applicable to the specific products tested.

## 5. Conclusion

• Self‐adhesive resin cement provided higher bond strength than GI cement.
• Sandblasting significantly increased the bond strength.
• Crown type had no significant effect.

It is suggested for practitioners to utilize self‐adhesive resin cement and sandblasting to improve bond strength between ZCs and molar bands.

## Funding

No funding was received for this manuscript.

## Conflicts of Interest

The authors declare no conflicts of interest.

## Data Availability

Data available upon request from authors.
